# Polyinosinic Polycytidylic Acid (poly I:C) Induces Neuronal Cell Death Through NF-κB-Mediated Inflammation in Human Microglia and Neuroinflammation-Induced Cognitive Impairment in Mice

**DOI:** 10.1007/s12035-025-05299-1

**Published:** 2025-11-19

**Authors:** Aseel Y. Altahrawi, Antonisamy William James, Zahoor A. Shah

**Affiliations:** 1 Department of Pharmacology and Experimental Therapeutics, College of Pharmacy and Pharmaceutical Sciences, The University of Toledo, Toledo, OH 43614 USA; 2 Department of Medicinal and Biological Chemistry, College of Pharmacy and Pharmaceutical Sciences, The University of Toledo, Toledo, OH 43614 USA

**Keywords:** Poly (I:C), Neuroinflammation, Proinflammatory cytokines, Neuronal death, Memory impairment

## Abstract

Neuroinflammation and neuronal death are direct consequences of persistent microglial activation, a phenomenon observed in many chronic neurological conditions. Activated microglia impact neuronal cells by releasing proinflammatory cytokines and inflammatory mediators, leading to neuronal damage and neurodegeneration. To investigate whether polyinosinic polycytidylic acid (poly I:C), a synthetic double-stranded RNA molecule, induces neuroinflammation and neuronal death, we exposed human microglia (HMC-3 cells) to poly I:C for 24 h, and performed inflammatory cytokine analysis. Additionally, to investigate whether poly (I:C) induces memory impairment and motor coordination deficits in mice, we conducted a behavioral assessment and also measured the expression of inflammatory cytokines in the brain. Poly (I:C) exposure significantly increased the mRNA and protein expression of inflammasome, proinflammatory cytokines (TNFα, IL-6, IL-1β, IL-8, IL-12, and IL-18) and chemokines in microglia. Poly (I:C) also significantly increased the translocation of NF-kB from the cytosol to the nucleus. Furthermore, the conditioned medium from poly (I:C)-treated cells markedly increased apoptosis in human neuronal cells (differentiated SHSY5Y cells) by activating pro-apoptotic markers, including Bax, Bad, cleaved caspase-3, cleaved PARP, and AIF. Mice exposed to poly (I:C) showed a significant increase in mRNA expression of inflammatory cytokines, such as IL-6 and TNF-α, in the hippocampus. A decrease in the percentage of alternation on the T-maze, reduced distance travelled, and average speed in the open field test indicate cognitive deficits as well as anxiety-like behavior in mice exposed to poly (I:C). These findings suggest that poly (I:C) induces neuroinflammation through the inflammasome and proinflammatory mediators via the NLRP3/NF-κB signaling pathway in vitro and in vivo.

**Supplementary Information:**

The online version contains supplementary material available at 10.1007/s12035-025-05299-1.

## Introduction

Neuroinflammation has recently emerged as a multidisciplinary field of research, focusing on the complex interaction between the immune system and the central nervous system (CNS). Neuroinflammation is the activation of the brain’s innate immune system when exposed to different inflammatory stimuli [1]. It is characterized by various cellular and molecular alterations inside the brain in which proinflammatory cytokines and other inflammatory mediators play a central role in regulating the process [1, 2]. Microglia, the brain’s resident macrophages, play a vital role in the brain’s innate immunity and homeostasis, as well as the pathogenesis of different neurological disorders [3]. When microglia are exposed to potentially harmful signals such as neurodegenerative debris, cellular stress, and injury, microglia become activated, proliferate, and transform from homeostatic microglia to phagocytic microglia [4]. Upon activation, microglia initiate a cascade of responses that initially protect the brain. However, prolonged activation leads to the excessive production of multiple inflammatory cytokines, ultimately resulting in neuronal dysfunction and neuronal death [5, 6].

Microglia act as a first line of defence against pathogens that invade the CNS. Different retroviruses (e.g., West Nile virus, Japanese encephalitic virus) cause encephalitis by producing dsRNA as they replicate within the CNS [7]. In neurotropic viral infections, microglia utilize pathogen recognition receptors, such as toll-like receptors (TLRs), to identify pathogen-associated molecular patterns (PAMPs) associated with invading viral pathogens [8]. Among these receptors, TLR3 plays a crucial role in the innate immune response to viral infections, as it primarily recognizes dsRNA, which is produced during viral replication [9]. TLR3 is highly expressed in microglia and detects dsRNA, activating downstream signaling pathways that promote the generation of various proinflammatory cytokines and type 1 interferon, which are critical for antiviral defence [10]. However, TLR3 has been demonstrated to mediate host-detrimental immunity. For example, mice lacking TLR3 showed greater resistance to infection with several viruses, such as Punta Toro, vaccine virus, and influenza virus, which is assumed to be due to TLR-3-mediated overproduction of inflammatory mediators [11–13].

Polyinosinic-polycytidylic acid (poly I:C) is a synthetic dsRNA molecule used in several preclinical models to simulate the replication of intermediates found in cells infected with viral RNA through the activation of TLR-3 receptors [14, 15]. Many studies have stimulated TLR-3 with poly (I:C), which is considered more potent than smaller viral dsRNA fragments obtained from viral preparations [15]. Nevertheless, recognizing dsRNA is only one aspect of the innate immune response to viral infection [16]. Poly I:C has been utilized to simulate the acute phase of viral infection, as it strongly activates type I interferons α and β, as well as other inflammatory cytokines [14]. Systemic administration of poly (I:C) accelerates neuronal loss in the substantia nigra pars compacta and striatum induced by 6-hydroxydopamine and paraquat [16, 17]. Furthermore, poly (I:C) administration activates the TLR3/tank-binding kinase 1/interferon-β signaling cascade and induces proinflammatory responses in RAW264.7 macrophages, thereby mimicking a viral infection [18]. Poly (I:C) injection upregulated COX-2 and microsomal prostaglandin E synthase-1 (PGES-1) expression, thereby increasing PGE2 synthesis in primary rat microglia through the mitogen-activated protein kinase, PI3K/mTOR, and NF-κB signaling pathways [19].

While HMC3 cells have been used to study neuroinflammatory responses, their inflammatory profile upon poly (I:C) stimulation has not been systematically examined. Here, we investigated the immune response of human microglial cells against poly I:C stimulation, with a particular focus on vital cytokines and the inflammasome at both the gene and protein levels. We also investigated microglia–neuron interactions to mimic in vivo neuroinflammation by treating neuronal cells with a conditioned medium obtained from poly I:C-activated microglia and measuring cytokines and apoptotic markers. Finally, we exposed mice to poly I:C to understand its effect on neuroinflammation and cognitive impairment.

## Methods

### Reagents

Poly (I:C) (Sigma-Aldrich, St. Louis, MO, USA, #P1530-100 mg) was dissolved at a concentration of 10 mg/mL in 0.9% NaCl by warming the mixture to 50 °C and then cooling it slowly to room temperature, allowing for reannealing. After that, poly (I:C) was aliquoted and stored at − 80 °C before use. LPS from *Escherichia coli* (Sigma-Aldrich, #L6529-1 mg) was solubilized in sterile water at a concentration of 1 mg/mL (stock solution), aliquoted, and stored at − 20 °C.

### Cell Culture

The HMC-3 cells, a human microglia clone-3 cell line (ATCC®, Manassas, VA, USA), were maintained in Eagle’s Minimum Essential Medium (EMEM) (ATCC®, Cat. No. 30‐2003) supplemented with 1% penicillin–streptomycin (PS) and 10% fetal bovine serum (FBS) (Gibco BRL, Grand Island, NY, USA). The cells were maintained in a 37 °C incubator with 5% carbon dioxide (CO_2_). Cells were seeded at 100,000 cells per well in six-well plates and allowed to grow for 72 h before treatment. Poly I:C treatment was conducted for 24 h, and LPS was used as a positive control.

### Cell Viability Assay

HMC-3 cells were seeded in 96-well plates at a density of 5000 cells per well. After 72 h, cells were treated with different concentrations of poly (I:C) (5, 10, 15, 20, 25, 50, 75, and 100 μg/mL) for 24 h. After 24 h, 10 µl of cell counting kit (CCK) reagent (Dojindo, Kumamoto, Kyushu, Japan, #CK04-11) was added to each well. The plate was incubated at 37 °C for 1 h, and then the absorbance was measured at 450 nm using a microplate reader. All readings were normalized to the average control value to represent the extent of cell mortality as a percentage.

### RNA Isolation and RT-qPCR

RNA was isolated from HMC-3 cells and brain tissue using TRIzol reagent (Invitrogen, Carlsbad, CA, USA, #15,596,026), according to the manufacturer’s instructions. Then, RNA’s concentration and purity were measured at 260 nm and 280 nm using an Epoch BioTek microplate reader (ELx800, BioTek, Winooski, USA). Two micrograms of RNA was reverse transcribed into single-stranded cDNA using a high-capacity cDNA reverse transcription kit (Applied Biosystems™, Waltham, MA, USA, Cat. No. 4368813). Specific transcripts were measured using RT-qPCR. cDNA was diluted 1:15 with nuclease-free water before use, then 10 µl of the diluted cDNA was mixed with 3 µl of forward and reverse primer (1.5 µl each) and 7 µl of SYBR Green Master mix (Applied Biosystems™, Waltham, MA, USA). The PCR process involved an initial denaturation at 95 °C for 10 min, followed by 40 amplification cycles of 15 s at 95 °C, 60 s at a uniform annealing temperature of 58 °C for all primer sets, and 15 s at 95 °C. A 5-min extension step was carried out at 72 °C. Amplification was performed using the ABI 7500 system (Applied Biosystems). Each mRNA expression analysis was performed in triplicate, and relative gene expression was estimated using the 2^−∆∆CT^ method, as described by James et al. [20]. The relative abundance of gene expression was normalized to β-actin as a housekeeping gene. The sequences of the primers are listed in Table 1.

**Table 1:** Representative table of primer sequence for PCR

Gene	Forward primer	Reverse primer
hIL-1β	AGCTACGAATCTCCGACCAC	CGTTATCCCATGTGTCGAAGAA
hTNF-α	GCTGCACTTTGGAGTGATCG	GTTTGCTACAACATGGGCTACAG
hIL-6	ACTCACCTCTTCAGAACGAATTG	CCATCTTTGGAAGGTTCAGGTTG
hIL-18	CGCTTCCTCTCGCAACAAAC	CCAGGTTTTCATCATCTTCAGC
hIL-8	ACTGAGAGTGATTGAGAGTGGAC	AACCCTCTGCACCCAGTTTTC
hIL-12	CAGAAGGCCAGACAAACTCT	GGTCTCTCTGGAATTTAGGCA
hCox-2	GCCAAGCACTTTTGGTGGAG	GGGACAGCCCTTCACGTTAT
hNLRP3	GCTGGCATCTGGATGAGGAA	GCCATCTTGACCCATCAGCA
hNox4	AAATGCACCAACAAATGGGG	CTGCTAGAGGCCCATGTAGAC
hCCL-2	ACAAGCAAACCCAAACTCCG	AACAGGGTGTCTGGGGAAAG
hCXL-16	TCTCAAAGAATGTGGACATGC	CAGGGGTGTGGATATCTGAA
hβ- actin	CATGTACGTTGCTATCCAGGC	CTCCTTAATGTCACGCACGAT
mIL-1β	GAAATGCCACCTTTTGACAGTG	TGGATGCTCTCATCAGGACAG
mTNF-α	CAGGCGGTGCCTATGTCTC	CGATCACCCCGAAGTTCAGTAG
mIL-6	CTGCAAGAGACTTCCATCCAG	AGTGGTATAGACAGGTCTGTTGG
GADPH	GCGAGACCCCACTAACATCA	GGCGGAGATGATGACCCTTT

### Immunocytochemistry

HMC-3 cells were cultured on glass coverslips precoated with poly-D-lysine (Gibco, BRL, Grand Island, NY, USA, #A3890401) in a six-well plate under conditions like those described previously. The cells were then treated with poly (I:C) (100 μg/mL) or LPS (100 ng/mL) and fixed with fresh 4% paraformaldehyde for 10 min before being washed with cold PBS. The fixed cells were permeabilized using 0.25% Triton X-100 (Sigma-Aldrich, #50–165–7277). After permeabilization, cells were washed with PBS before 30-min incubation with blocking serum (1% BSA and 22.52 mg/ml glycine dissolved in 0.1% Tween 20 and PBS), followed by overnight 4 °C incubation in primary antibody (rabbit anti-NF-κB P65 (Cell Signalling Technology, Danvers, MA, USA #8242), 0.7:1000). After washing with PBS, cells were incubated for 1 h at room temperature with Alexa Fluor® goat Anti-rabbit IgG secondary antibody (1:1000; Cell Signalling Technology, #4412). Coverslips were washed with PBS three times and then mounted with DAPI (Invitrogen, Carlsbad, CA, USA, #00–4959-52) on microscope slides. Images were captured with a Nikon TE2000-U fluorescence microscope, and the fluorescence intensity was analyzed with Nikon Imaging Software (NIS Elements).

### Subcellular Fractionation

Cells were washed with cold PBS, and then 100 μL of cytosol lysis buffer was added to each well (20 mM HEPES PH = 7.2, 10 mM KCl, 2 mM MgCl_2_, 0.5% NPO_4_, 1 mM Na_3_Vo_4_, and phosphatase and protease inhibitor cocktail, 10 µL/1.0 ml). The cells were then collected in a 1.5-ml eppendorf tube, vortexed for 30 s and incubated on ice for 30 s and repeated for at least ten cycles and centrifuged at 10,000 × *g* for 15 min at 4 °C. The resulting supernatant was collected as a cytosolic fraction. The nuclear pellet was washed twice with 300 μL of the cytosolic lysis buffer and resuspended in an appropriate volume of RIPA buffer to extract the nuclear proteins. The extract was then centrifuged at 10,000 × *g* for 10 min and stored as the nuclear fraction.

### Conditional Media

SH-SY5Y, human neuroblastoma cells (1 × 10^5^), were seeded in six-well plates and cultured in Dulbecco’s Modified Eagle’s Medium and Ham’s F-12 Nutrient Mixture (DMEM F12, 10% FBS and 1% PS) for 4 days at 37 °C and 5% CO_2_ incubator. SH-SY5Y cells were differentiated into neuronal cells by culturing in 10 µM retinoic acid for 4 days, and cells were treated with conditioned medium collected from HMC3 cells for 24 h. Simultaneously, to induce the proinflammatory cytokine secretion in the medium, HMC3 cells were treated with poly (I:C) 250 µg/ml for 6 h. After 6 h, the medium was removed and replaced with fresh EMEM medium without poly (I:C) and cultured for 24 h. The medium was collected from HMC3 cells treated with poly (I:C) and added to differentiated neuronal cells to determine the effect of poly (I:C)-induced inflammatory cytokines on neuronal death. For cell viability, assay (CCK-8), SHSY5Y cells were seeded (5 × 10^3^) in 96-well plates and differentiated into neuronal cells as described previously. The culture medium was replaced with conditioned medium collected from HMC3 cells treated with poly (I:C), to assess the effect of poly (I:C)-induced inflammatory cytokines on the viability of neuronal cells.

### Western Blotting

Cells were washed with PBS, lysed with RIPA buffer (ThermoFisher Scientific, Waltham, MA, USA, #89,901), mixed with protease and phosphatase inhibitor cocktail (ThermoFisher Scientific, #PI78441), and incubated on ice for 20 min. The protein concentration in the sample was measured using Bradford reagent (Bio-Rad Laboratories, Hercules, CA, USA, #5,000,006) according to the manufacturer’s instructions. The protein samples were denatured at 95 °C for 7 min; 40 µg of protein was loaded onto a 12% SDS–polyacrylamide gel and then underwent electrophoresis for approximately 90 min. Then, the proteins were transferred to a PVDF membrane using a wet transfer system. After that, the membranes were blocked with 1% BSA (1 × TBST) for 20 min to prevent non-specific binding. The membranes were incubated with different primary monoclonal antibodies, including IL-1β, IL-6, TNF-α, NLRP3, Casp-3, C-cas3, BAD, BAX, and β-actin (cell signalling technology) overnight at 4 °C; β-actin was used as a loading control. The next day, membranes were washed with TBST (1 × Tris-buffered saline + Tween 20) three times, 10 min each, and then incubated with secondary horseradish peroxidase (HRP)-linked antibodies for 2 h at room temperature. The protein bands were quantified using Image-J software 1.53 (National Institutes of Health, Bethesda, MD, USA) and normalized with β-actin and presented as relative expression to control.

### Animals

Eight to twelve-week-old C57BL/6 male mice weighing 25–28 g were housed in plastic cages (5 mice/cage) with free access to food and water. The temperature was maintained at approximately 22 °C with a 12:12-light–dark cycle. Animals were allowed to acclimate for 3 days before starting the experimental protocol. The experimental protocol (UT-108877) was approved by the Institutional Animal Care and Use Committee (IACUC) at The University of Toledo. Mice were randomized into two groups (*n* = 5 each), including a control and a poly (I:C) group. The control group received the vehicle (0.9% NaCl) IP for three doses. The poly (I:C) group was treated with poly (I:C) (20 mg/kg/day) on alternate days, and a total of three doses were administered before undergoing behavioral tests and finally euthanization. The cortex and hippocampus regions of the brain were isolated, and the inflammatory cytokines were analyzed by QRT-PCR as described previously.

### Neurobehavioral Tests

Mice underwent behavioral tests beginning on the final poly (I:C) injection day, at a rate of two tests per day over two consecutive days, following this sequence: open field (OF) followed by T-maze on the first day, and the rotarod and grip strength tests on the second day. All the behavioral tests were conducted by an individual blinded to the experimental groups.

The OF test is used to assess locomotor activity, exploration, and anxiety-like behavior in mice. Briefly, mice were placed in open-topped white crystal square boxes and allowed to explore freely for 30 min. The total distance travelled, average speed, and center zone activity were recorded using an overhead camera linked to a computer with an ANY-maze tracking system.

The T-maze test was employed to assess the mouse’s spatial memory. Mice were placed in a T-shaped maze comprising one start arm and two goal arms. The mice underwent three trials, with each trial consisting of two sessions. In the first session, the mice were placed in the start arm and then confined for 30 s after selecting one of the goal arms. The second session began with the mouse being returned to the starting arm, facing away from the goal arms, and given the choice to select one of the goal arms. The percentage of spontaneous alteration rate was determined as prescribed previously [21].

Rotarod was used to evaluate motor coordination and balance after PIC injection. The device was designed to initially rotate at a speed of 4 rpm and then increase by 10 rpm every 10 s, reaching a maximum of 40 rpm in 300 s. Each mouse underwent three trials with a 5-min gap between trials. Each trial begins by placing the mouse on the rod and calculating how long it takes for the mouse to fall. The average duration spent on the rotating rods was calculated for each mouse.

Finally, the grip strength test was performed to assess the strength of neuromuscular function following the injection of poly (I:C). The mice were positioned into the grip bar and allowed to hold it spontaneously. The force required to detach the mouse from the bar, as it appeared on the digital screen, was recorded as a measure of its muscle strength.

### Statistical Analyses

Data represent at least three independent experiments, with at least two to three replicates each. Data are presented as the mean ± standard error of the mean (SEM). Statistical analyses were performed using the GraphPad Prism (9.3.1) program. For comparisons among multiple groups, normally distributed data were analyzed using one-way analysis of variance (ANOVA), followed by a Bonferroni post-hoc test for multiple comparisons. The Welch ANOVA test was used to analyze the data with unequal variances, while the Kruskal–Wallis test was used to analyze the data that deviated from normality. For comparisons between two groups, the unpaired *t* test was used for normally distributed data with equal variances, Welch’s *t* test for unequal variances and the Mann–Whitney *U* test for non-normally distributed data. *P* values less than 0.05 were considered statistically significant.

## Results

### Poly (I:C) Exposure Reduced Microglial Viability

**Fig. 1:**
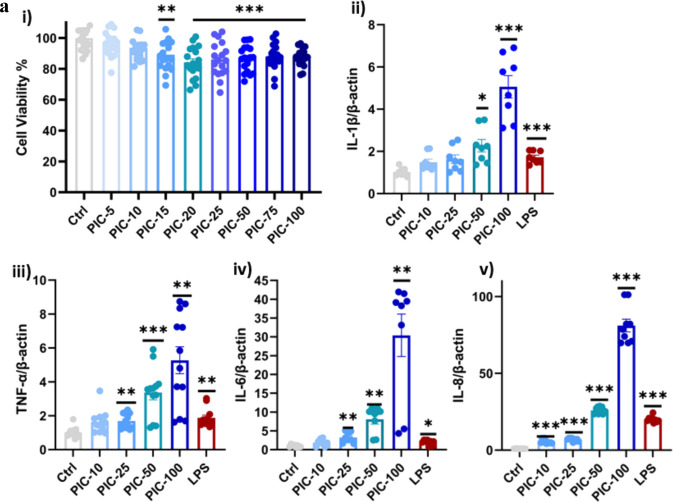

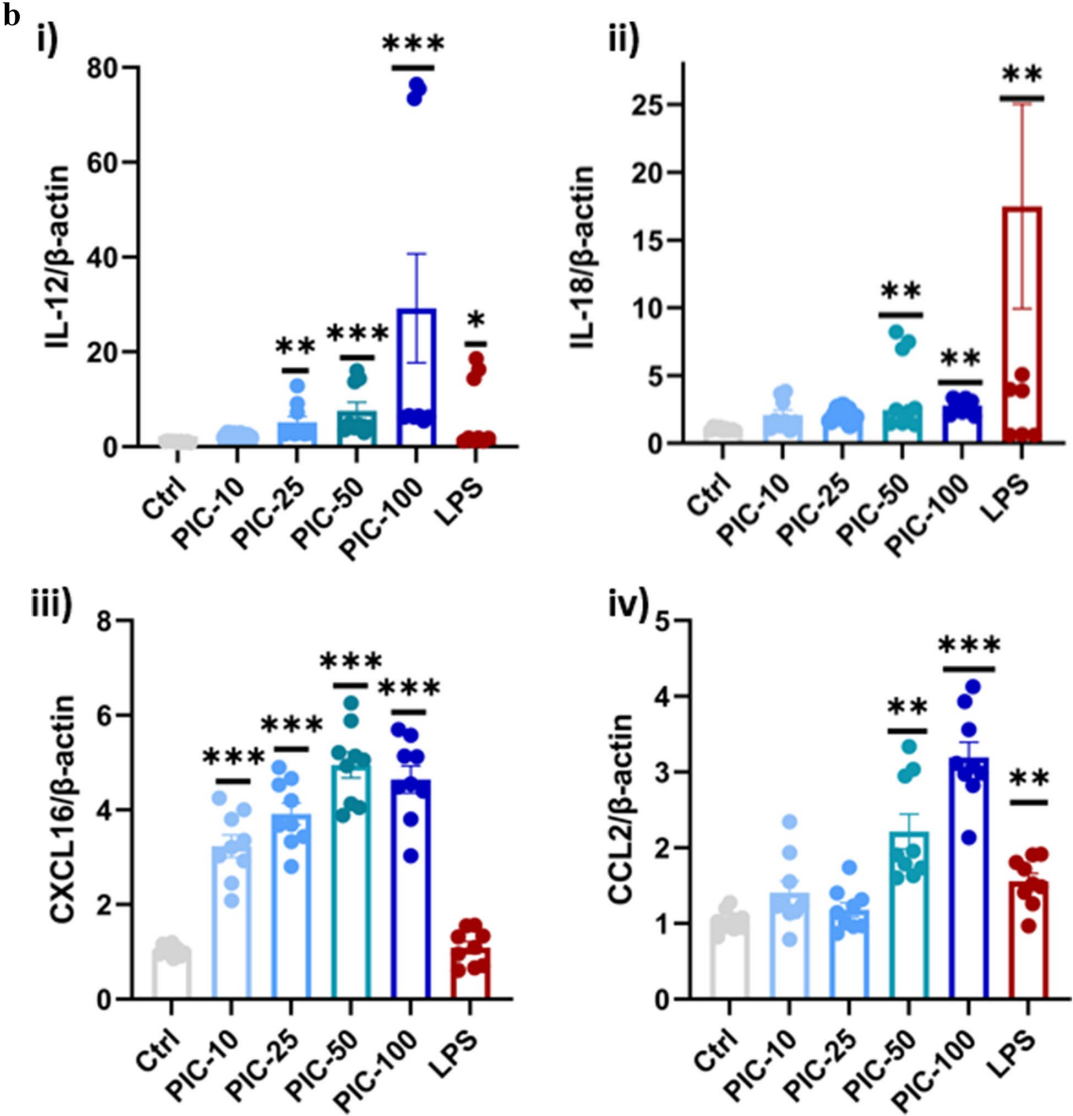

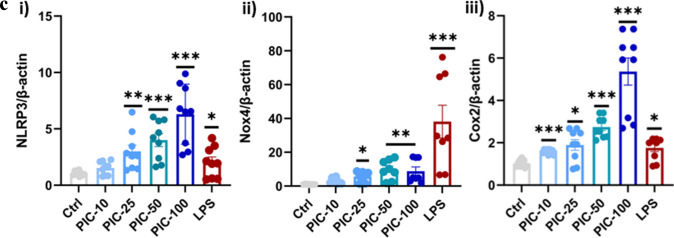
**a** Poly (I:C) reduced microglia viability and inflammation. HMC-3 cells were seeded in 96-well plates and treated with varying concentrations of poly (I:C). Cell viability was measured using the CCK-8 assay kit, as described in the Methods section. (ii–v) The relative mRNA expression levels of different cytokine genes were determined in poly (I:C)-treated HMC-3 cells and calculated using the 2^−ΔΔCT^ method. β-actin was used as the housekeeping gene for normalization. Data are presented as mean ± standard error of the mean (SEM), *n* = 3. Significant differences are indicated as follows: *P < 0.05, **P < 0.01, ***P < 0.001. **b** Poly (I:C) increased the inflammatory cytokines and chemokines. HMC-3 cells were seeded in 6-well plates and treated with poly (I:C). The relative mRNA expression levels of different cytokines and chemokines were determined in poly (I:C)-treated HMC-3 cells and calculated using the 2^−ΔΔCT^ method. β-actin was used as the housekeeping gene for normalization. Data are presented as mean ± standard error of the mean (SEM), *n* = 3. Significant differences are indicated as follows: *P < 0.05, **P < 0.01, ***P < 0.001. **c** Poly (I:C) increased the inflammasome marker, as well as oxidative stress markers. HMC-3 cells were seeded in six-well plates and treated with poly (I:C) and LPS for 24 h. (i–iii) The relative mRNA expression levels of different inflammasome and oxidative stress-related genes were determined in poly (I:C)-treated HMC-3 cells and calculated using the 2^−ΔΔCT^ method. β-actin was used as the housekeeping gene for normalization. Data are presented as mean ± standard error of the mean (SEM), *n* = 3. Significant differences are indicated as follows: *P < 0.05, **P < 0.01, ***P < 0.001

We used the CCK-8 assay to evaluate the response of HMC-3 cells stimulated with poly (I:C) over a 24-h period. Initially, HMC-3 cells were seeded in 96-well plates and grown in a standard cell culture medium. The cells were then treated with different concentrations of poly (I:C) (5–100 μg/mL), and the percentage of viability was compared to that of the control group. As shown in Fig. 1a (i), there was a dose-dependent effect on cell viability. At lower concentrations (5 and 10 μg/mL), cell viability remained similar to that of the control group (*P* > 0.05). However, at higher concentrations (15 to 100 μg/mL), viability was significantly reduced compared to the control group (*P* < 0.01). Therefore, we selected the 100 μg/ml concentration, which resulted in a 13–15% reduction in cell viability.

### Poly (I:C) Exposure Upregulated Gene Expression of Inflammatory Cytokines

HMC-3 cells were treated with increasing concentrations of poly (I:C) (10 to 100 μg/mL), and LPS (100 ng/mL) was used as a positive control, which induced a dose-dependent increase in inflammatory cytokines. Significant increases (*P* < 0.05) were observed in IL-1β, TNF-α, IL-6, and IL-8 levels compared to the negative control group (untreated group), with the highest expression seen at 100 μg/mL (Fig. 1a (ii–v)). Moreover, the significant upregulation of IL-18 and IL-12 (Fig. 1b (i–ii)), two key mediators of Th1-mediated immunity, also suggests that antiviral and neuroinflammatory responses were activated. Except for IL-18, poly (I:C)-induced cytokine levels are equivalent to or even more significant than those caused by LPS, indicating its potent immune-activating properties.

CCL2 and CXCL-16, critical chemokines associated with leukocyte recruitment and BBB dysfunction, were also upregulated in response to poly(I:C) treatments. Interestingly, CXCL16 expression was significantly elevated by the lowest concentration of poly (I:C) used (*P* < 0.001; Fig. 1b (iii)), indicating an early and strong chemotactic response. CCL2 expression remained unchanged at concentrations of 10 and 25 μg/ml. But it dramatically increased at higher concentrations of poly (I:C) at 50 and 100 μg/ml (*P* < 0.01), indicating a dose-dependent activation of monocyte recruitment (Fig. 1b (iv)).

### Poly (I:C) Exposure Increased Gene Expression of the Inflammasome and Oxidative Stress

We investigated whether poly(I:C) induces NLRP3 expression, a marker of the inflammasome, as both IL-1β and IL-18 are regulated by inflammasome activation. Poly (I:C) treatment significantly upregulated NLRP3 mRNA expression in a dose-dependent manner (*P* < 0.01; Fig. 1c (i)), indicating that the inflammasome was activated.

To further investigate oxidative stress, we measured the expression of NOX-4 and COX-2, which are significantly increased in response to poly (I:C) treatment (*P* < 0.05; Fig. 1c (ii and iii)). However, despite this increase, even the highest concentration of poly (I:C) failed to increase NOX4 expression to a level comparable to that of LPS, demonstrating that poly (I:C)-induced oxidative stress may be less severe than LPS-induced oxidative responses. The concurrent elevation of COX-2 expression, a critical enzyme in the arachidonic acid pathway, indicates an increase in proinflammatory lipid mediators resulting in further exacerbation of neuroinflammation.

### Poly (I:C) Exposure Increased Protein Expression of the Inflammasome and Inflammatory Cytokines

**Fig. 2:**
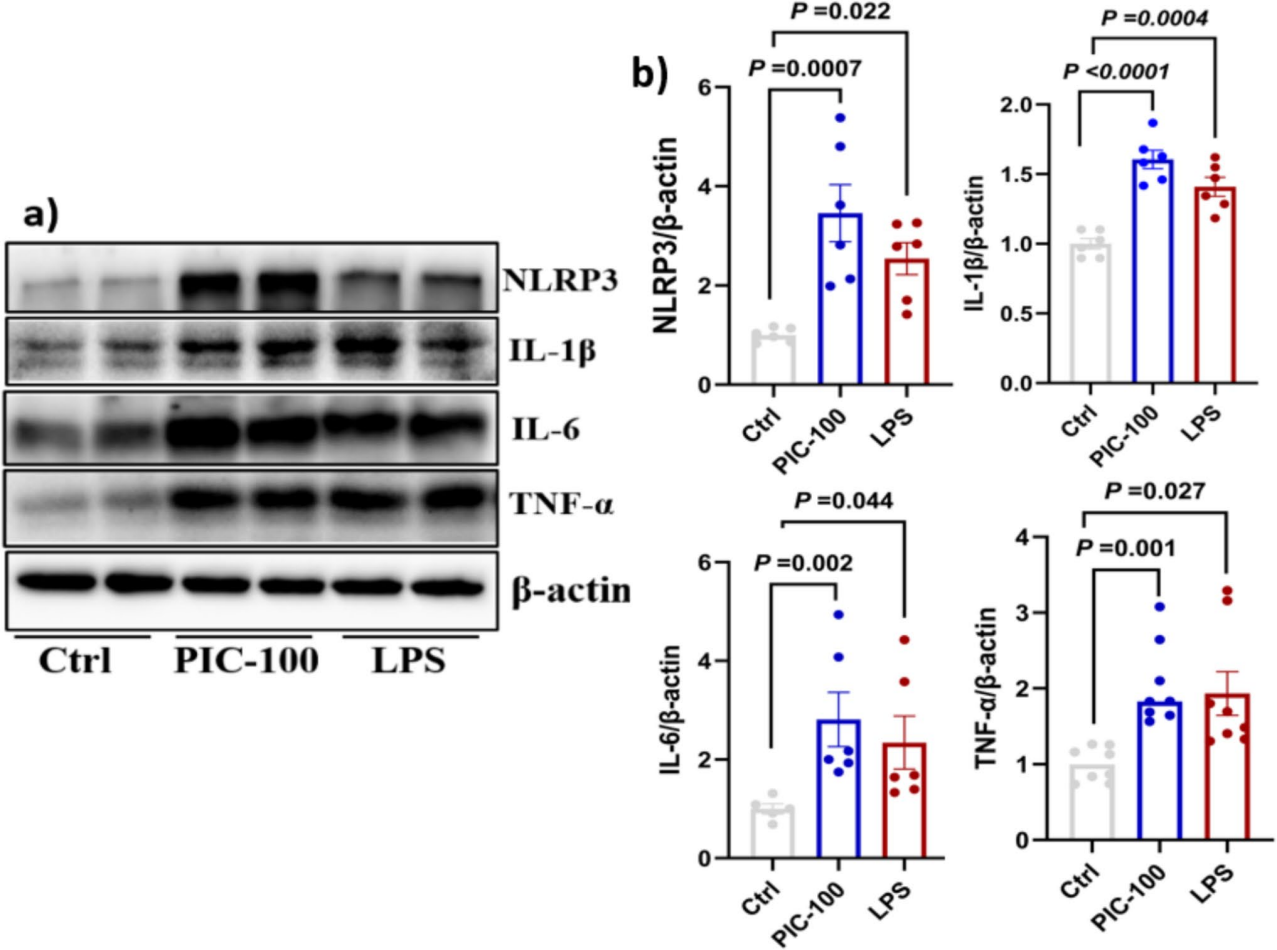
Poly (I:C) elevates inflammatory cytokines (IL-6 and TNFα) and inflammasome markers (NLRP3 and IL-1β). **a**, **b** HMC3 cells were seeded in six-well plates and treated with poly I:C and LPS. Western blotting was performed to assess protein expression, and the bands were quantified using ImageJ software and normalized to β-actin. The relative protein expression data represent the means of three independent experiments ± standard error of the mean (SEM), *n* = 3

The protein expression levels of IL-6, IL-1β, TNF-α, and NLRP3 were assessed by Western blotting in response to treatment with poly (I:C) and LPS. In the control group, all these inflammatory markers were expressed at minimal baseline levels. LPS significantly increased the expression of these markers, including IL-6 (2.3-fold), IL-1β (1.4-fold), TNF-α (1.93-fold), and NLRP3 (2.5-fold) compared to the control (*P* < 0.05), indicating activation of the NF-κB and NLRP3 inflammasome pathways. Poly (I:C) also induced a significant increase in inflammatory markers compared to the control: IL-6 increased by approximately 2.8-fold, IL-1β by 1.6-fold, TNF-α by 1.7-fold, and NLRP3 by 3.5-fold (*P* < 0.01; Fig. 2a and b).

### Poly (I:C) Increased the Protein Expression of Nuclear NF-κB

**Fig. 3:**
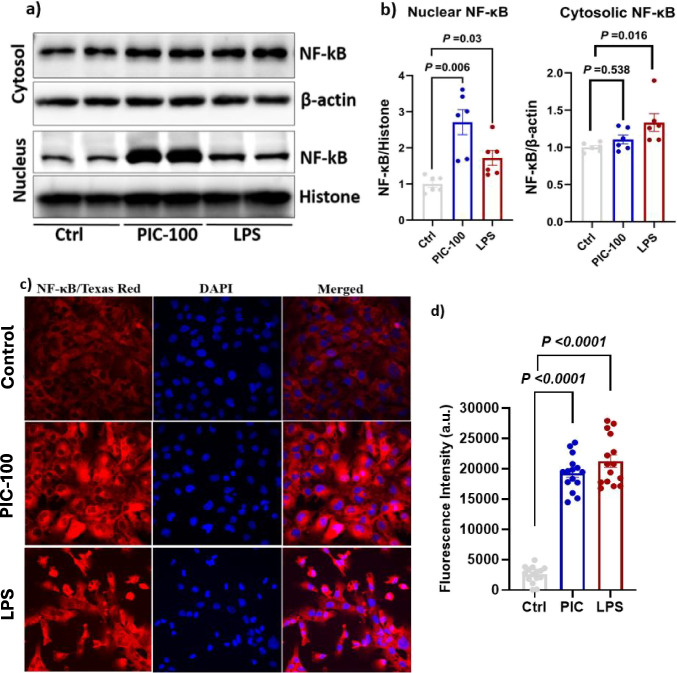
Poly (I:C) increased the nuclear localization of NF-kB in HMC-3 cells. **a**, **b** Nuclear and cytosolic fractions were isolated, and Western blotting was performed to assess NF-κB expression. Protein levels were normalized to histone for nuclear fractions and β-actin for cytosolic fractions, as described in the Methods section. **c**, **d** HMC3 cells were seeded on poly-D-lysine-coated coverslips in six-well plates and treated with poly (I:C) and LPS. Immunocytochemistry was performed as described in the Methods section. NF-κB was detected using a Texas Red-conjugated secondary antibody, and nuclei were stained with DAPI. **c** Fluorescence imaging was performed at 40 × magnification using a Nikon TE2000-U fluorescence microscope. **d** The relative fluorescence intensity of NF-κB protein expression was measured from three biological replicates. Data represent the means of three independent experiments ± standard error of the mean (SEM), *n* = 3

The nuclear translocation of NF-κB p65, the hallmark of NF-κB pathway activation, was evaluated by subcellular fractionation (Fig. 3). In the control cells, minimal nuclear NF-κB accumulation was observed, which is consistent with an inactive state. Poly (I:C) treatment at 100 μg/mL led to a significant increase in nuclear NF-κB levels (1.6-fold and 2.87-fold, respectively) compared to the control (*P* < 0.01; Fig. 3a and b). LPS-treated cells have also exhibited an extensive accumulation of nuclear NF-κB p65, accompanied by a significant increase in cytoplasmic NF-κB signaling (*P* < 0.05; Fig. 3b). These results were further supported by immunocytochemical analysis. As shown in Fig. 3c and d, in the control, NF-κB staining was predominantly located in the cytoplasm. Poly (I:C)-treated cells showed intensive and widespread nuclear localization, indicating a strong NF-κB activation, while LPS-treated cells showed a moderate increase in NF-κB nuclear localization. Both LPS and poly (I:C) increased NF-κB signalling, but poly (I:C)-induced nuclear translocation and activation were observed to be higher.

### Poly (I:C)-Treated HMC-3 Conditioned Media Decreased Cell Viability in Differentiated SH-SY5Y Cells

**Fig. 4:**
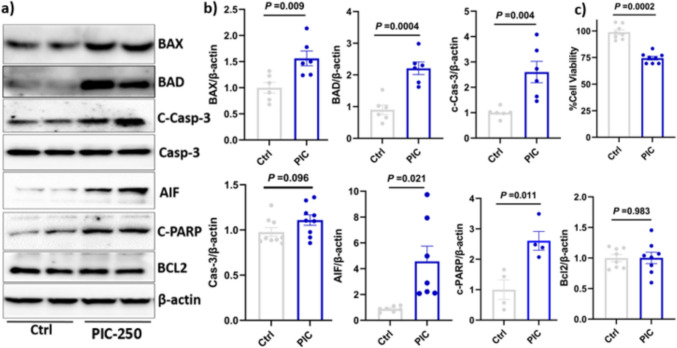
Poly (I:C)-induced inflammatory cytokine-mediated apoptosis in neuronal cells. **a**, **b** Protein expression of apoptosis markers and their relative quantification: neuroblastoma SH-SY5Y cells were differentiated into neuronal cells using 10 µM retinoic acid for 5 days. The differentiated cells were then treated with conditioned medium collected from HMC3 cells following 24 h of poly (I:C) treatment. Conditioned media from stimulated HMC-3 cells. **a**, **b** Protein expression of apoptosis biomarkers. **c** Cell viability assay. The relative protein expression data represent the means of three independent experiments ± standard error of the mean (SEM), *n* = 3

To evaluate the neurotoxic effect of poly (I:C)-induced inflammatory cytokines, differentiated SH-SY5Y cells were exposed to conditioned media obtained from HMC-3 cells after treatment with different concentrations of poly (I:C) (100, 150, 200, and 250 μg/mL) for 24 h. As shown in Fig. 4c, the CCK-8 assay showed a significant reduction in neuronal viability at a concentration of 250 μg/ml. Compared to the control, neuronal viability decreased by approximately 25% at 250 μg/mL (*P* < 0.05).

### Poly (I:C) Exposure Activated the Intrinsic Apoptosis Pathway in Neuronal Cells

To determine whether intrinsic apoptotic pathways contributed to the observed neurotoxicity, we measured the expression of key regulators in the intrinsic pathway. Protein expression results indicated a significant increase in proapoptotic signalling in SH-SH5Y cells exposed to conditioned media obtained from HMC-3 cells treated with 250 μg/mL (Fig. 4a, b). Bax protein expression increased by 1.7-fold (*P* < 0.05), whereas Bad protein expression increased by 2.2-fold (*P* < 0.01) compared to the control group. The expression of cleaved caspase-3 protein was significantly elevated, showing a 2.6-fold increase (*P* < 0.05). In contrast, total caspase-3 expression levels did not change significantly (*P* > 0.05), indicating that poly (I:C) promotes caspase-3 activation rather than affecting its total expression. To further investigate the downstream pathway of apoptosis, we observed a significant increase in the expression levels of cleaved PARP (c-PARP), 2.6-fold, which is consistent with increased execution-phase apoptosis and DNA damage. On the other hand, the expression of antiapoptotic protein BCL2 did not change significantly (*P* > 0.05). To further evaluate caspase-independent cell death, particularly mitochondrial-mediated apoptosis, we examined the expression of apoptosis-inducing factor (AIF). AIF expression was dramatically increased sixfold compared to the control group.

### Poly (I:C) Administration Induced Impairment in Spatial Memory and Increased Anxiety-Like Behavior, Without Affecting Motor Coordination and Muscle Strength

**Fig. 5:**
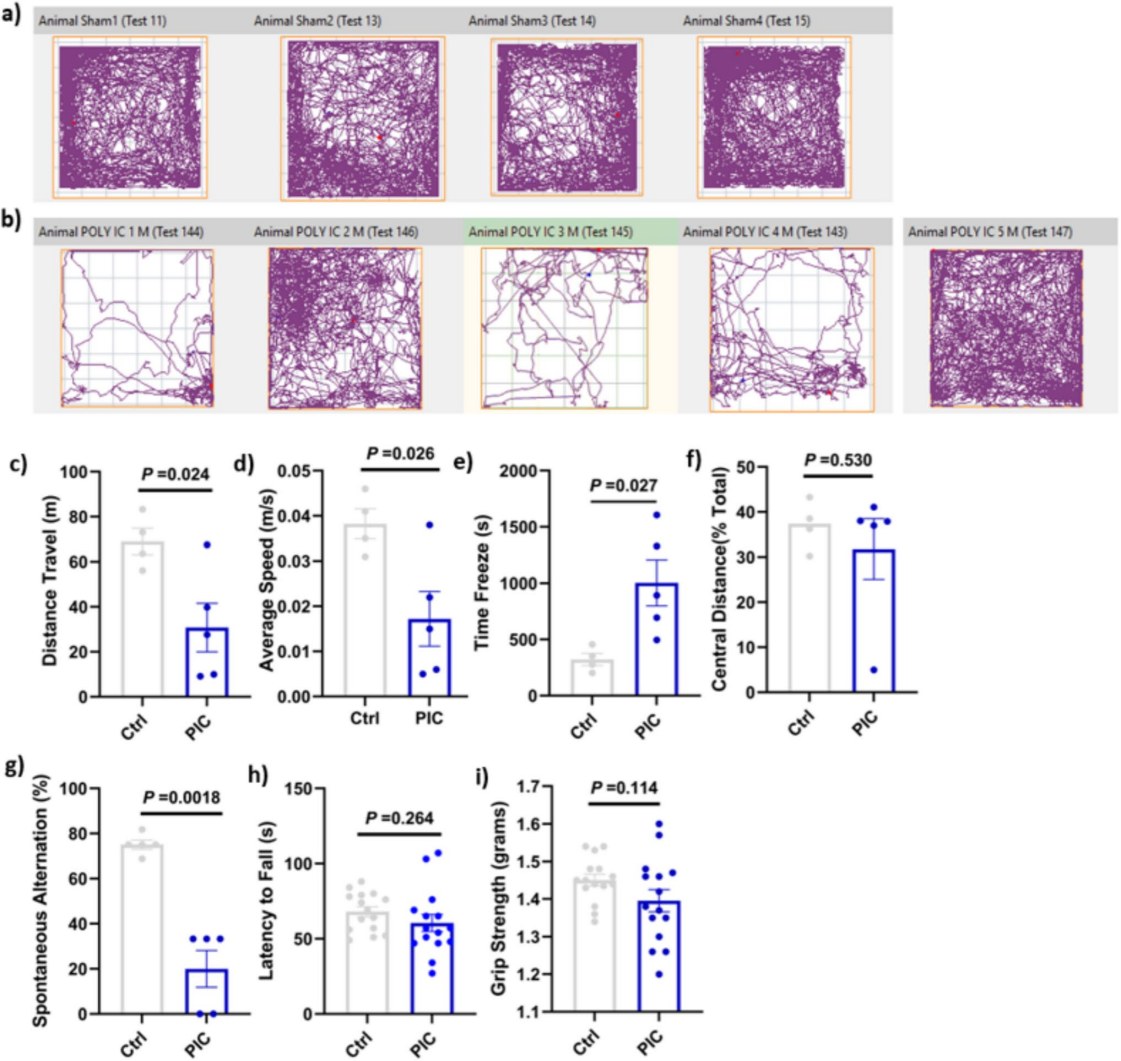
Poly (I:C) increased neuroinflammation and induced neurobehavioral dysfunction. Mice (*n* = 5) were injected poly (I:C) (20 mg/kg), intraperitoneally, and control mice (*n* = 4) were injected with PBS on alternate days in a week. On day 5, the T-maze and open field (OF) tests were performed, and on day 6, grip strength and rotarod tests were carried out. The animals were sacrificed afterwards. **a**, **b** Illustrative examples of control and poly (I:C)-treated mice’s travel pathway on the open field test (**c**), the total distance travelled (**d**), the average speed (**e**), and time freeze and central distance (**f**). The spontaneous alternation rate in the T-maze (**g**), the latency to fall (time in seconds) was measured in the rotarod (**h**), and the grip strength test (**i**). Data are presented as the means ± standard error of the mean (SEM). Statistical differences were determined by an unpaired *t* test

In the open-field test, poly (I:C)-treated mice exhibited a significant reduction in total distance travelled and average speed, demonstrating anxiety-like behavior. Poly (I:C)-treated mice also showed a more extended time immobile compared to the control group (*P* < 0.05; Fig. 5a–f), although no significant difference was observed in the central distance travelled. Additionally, animals exposed to poly (I:C) showed a significant reduction in spontaneous alternation in the T-maze test (*P* < 0.05), indicating a working memory deficit (Fig. 5g). In contrast, there was no significant difference in rotarod performance between groups, suggesting no impact on motor coordination (*P* > 0.05). Likewise, grip strength measurements remained unchanged, indicating no effect on muscle strength (*P* > 0.05; Fig. 5h and i).

### Poly (I:C) Triggered a Neuroinflammatory Response, Characterized by Elevated Levels of the Proinflammatory Cytokines in the Hippocampus but not in the Cortex

**Fig. 6:**
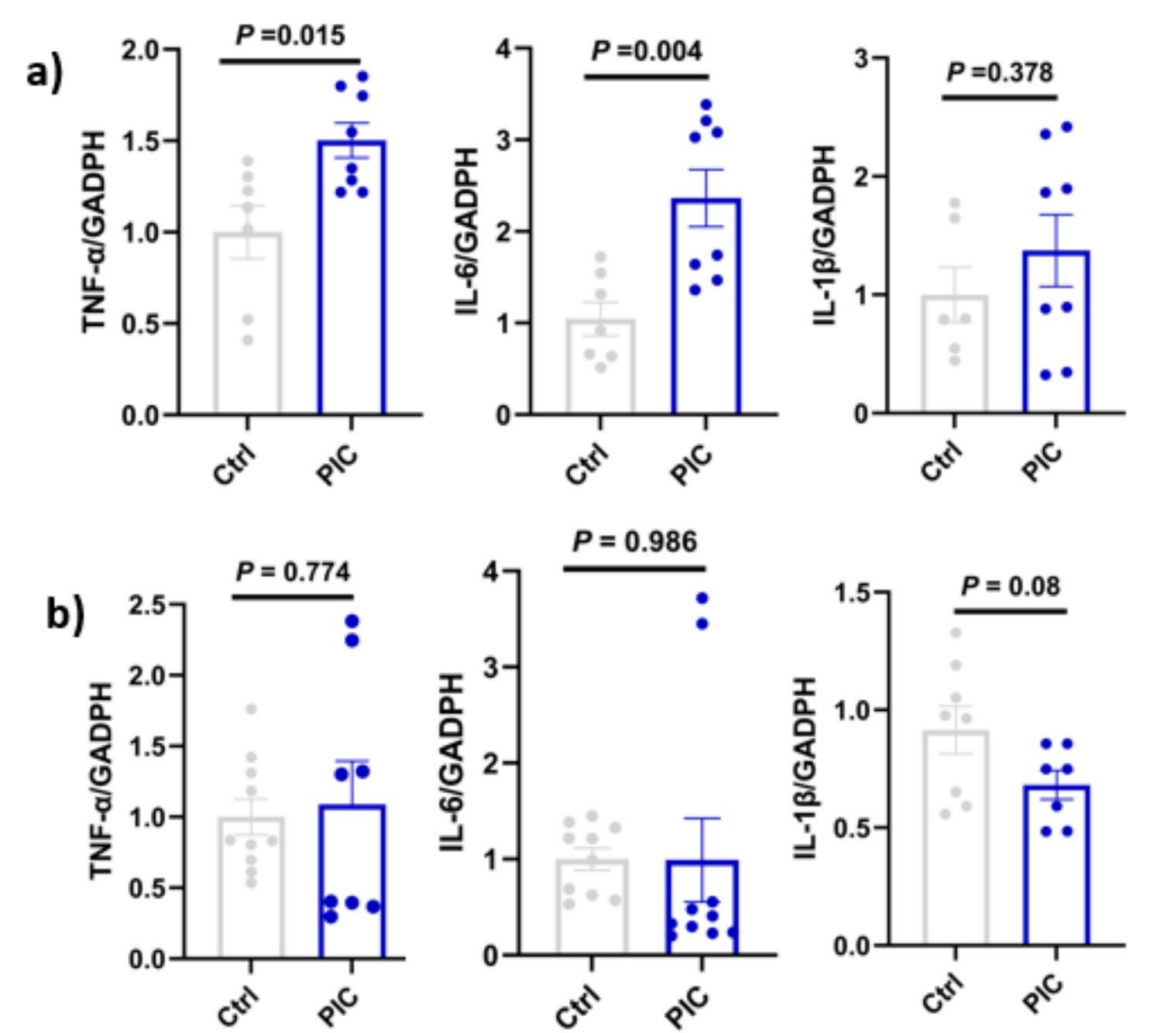
Poly (I:C) increased neuroinflammation in the hippocampus of mice. Mice (*n* = 5) were intraperitoneally injected with poly (I:C) (20 mg/kg) and control mice (*n* = 5) with PBS through on alternate days of the week. The cortex and hippocampus regions of the brain were isolated, and the inflammatory cytokines were measured by q/RT-PCR as described in the Method section. mRNA expression in the hippocampus (**a**) and cortex (**b**). Data represent the means ± standard error of the mean (SEM)

In response to poly (I:C) exposure, a 2.4-fold increase in IL-6 gene expression levels was observed in the hippocampus 24 h after the administration of the last dose compared to the vehicle-treated control (*P* < 0.05). A similar pattern of gene expression was observed in TNF-α, with a 1.5-fold increase in the hippocampus region of poly (I:C)-exposed animals compared to the vehicle-treated control (*P* < 0.05). In contrast, IL-1β exhibited an upward trend in its gene expression; however, this change was not statistically significant (*P* > 0.05; (Fig. 6a).

In the cortex, no significant change was observed in the mRNA expression levels of the previous cytokines (*P* > 0.05; Fig. 6b), suggesting that the cortical tissue was not affected by the treatment, which further confirmed the behavioral outcomes, indicating the preservation of motor performance.

## Discussion

In the present study, we observed that poly (I:C) induces a strong neuroinflammatory response that is comparable to, and even surpasses, that induced by LPS. In our in vitro study, we observed that poly (I:C) activated microglial cells, significantly increasing the expression of key inflammatory cytokines and chemokines, similar to those observed in microglia treated with LPS. Conditioned media collected from poly (I:C)-treated microglia also exhibited a detrimental effect on neuronal cells, resulting in reduced neuronal viability and activation of the intrinsic apoptotic pathway. In our in vivo study, the administration of poly (I:C) increased the expression of inflammatory cytokines in the hippocampus, resulting in a significant decline in cognitive function.

Viral infection affecting the brain and the spinal cord can have serious and even fatal consequences. Therefore, understanding the process by which the central nervous system effectively controls viral infections is essential for developing therapeutic agents and optimizing the time for initiating drug therapy [22]. Previous studies have investigated the impact of poly (I:C) on microglial activation and neuroinflammatory responses [3, 19, 23, 24]. It has been proven that poly (I:C) robustly activates microglia through TLR3 receptors, producing proinflammatory cytokines [3, 23]. Subsequently, this study was the first to evaluate the neuroinflammatory response of human microglial cells to poly (I:C) stimulation. The findings of this study consistently demonstrate that poly (I:C) induces a potent inflammatory response, as evidenced by the significant upregulation of key cytokines and inflammasome markers at both transcriptional and protein levels.

Poly (I:C) is a viral ds-RNA analogue that activates various immune cells through pattern recognition receptors such as TLR3 and melanoma differentiation-associated protein 5 (MDA5), which are expressed in microglia and act as viral RNA sensors [18]. The activation of these receptors triggers signalling cascades that activate NF-κB and interferon regulatory factors (IRF) [19]. In our study, poly (I:C) activation promotes classical inflammatory pathway signaling, as evidenced by the nuclear translocation of NF-κB. This nuclear translocation enhances the transcription of proinflammatory biomarkers, increasing the mRNA levels of several cytokines (e.g., IL-1β, IL-6, TNF-α, IL-12, and IL-18). These results were further confirmed by increased staining intensity in the poly (I:C)-exposed groups suggested a strong activation of the inflammatory pathway. To support our data, Wegrzyn et al. also demonstrated that poly (I:C) increases the production of TNF-α, IL-6, and IL-1β via TLR3 signaling in mouse primary microglia [23]. Notably, the response generated by poly (I:C) was comparable to that of LPS, a traditional TLR4 agonist. LPS treatment led to a significant increase in pro-inflammatory cytokines, confirming the efficacy of our model to induce neuroinflammation.

The observed increase in IL-12 and IL-18 gene expression suggests the activation of an innate immune response that promotes INF-γ production. IL-12 has been shown to activate STAT4 and promote IL-18R expression, making T and B cells more sensitive to IL-18 [25]. The IL12/IL18 synergistic effect promotes strong INF-γ production, consistent with poly (I:C) biological activity as a viral dsRNA analogue.

The increase in NLRP3, IL-1β, IL-6, and TNF-α was further validated by assessing protein expression levels. The magnitude of induction was comparable for TNF-α, but more intense than LPS for IL-6, IL-1β, and NLRP3. These findings suggest that the poly (I:C) mechanism of inflammasome activation may involve distinct upstream signals, potentially mediated by the TLR3/RIG-I-like receptor pathway. These cytokines are pivotal mediators of neuroinflammation, with IL-1β and TNF-α notably recognized for enhancing the inflammatory response through autocrine and paracrine signaling by stimulating macrophages to release more proinflammatory cytokines (e.g., IL-6 and IL-8), as well as reactive oxygen and nitrogenous species [26]. Several studies have proved that mitochondrial and NADPH oxidase-derived ROS promote the activation of NLRP3 inflammasome, and trigger caspase-1-dependent maturation of IL-18 and IL-1β [27, 28]. Particularly, NOX4 has been linked to redox signalling that regulates inflammasome activity [29], while COX-generated ROS may exacerbate this reaction, thus justifying the increased mRNA expression of IL-8, COX2, and Nox4.

Interestingly, our results demonstrated a significant increase in the gene and protein expression of NLRP3, characterized by its sensor protein (a pattern recognition receptor), which is oligomerized to create a pro-caspase 1 activation platform in response to PAMPS [30]. Activation of the NLRP3 inflammasome results in caspase 1-dependent maturation and secretion of IL-1β and IL-18 [31], and its upregulation in response to poly (I:C) treatment, along with IL-1β overexpression, suggests that poly (I:C) may activate signal 1 (priming), and may be an element of signal 2 (activation) in inflammasome pathway regulation. This finding is important as it links viral stimulation to both the transcription of proinflammatory cytokines and the assembly of innate immune sensors that enhance immune responses.

A sustained neuroinflammatory environment may accelerate neuronal cell senescence, promoting apoptosis under stressful conditions [32, 33]. For instance, it is well known that in neurodegenerative diseases such as Alzheimer’s and Parkinson’s, prolonged microglia activation induces neuronal death predominantly through the intrinsic pathway [34]. In this study, we treated neuronal cells with conditioned media collected from activated HMC-3 cells and found a significant increase in pro-apoptotic markers. This could be due to the vital role of soluble inflammatory mediators in facilitating neurotoxicity [35]. It has been demonstrated that elevation of Bax and Bad altered the Bcl2/Bax balance, favoring mitochondrial outer membrane permeabilization (MOMP) [36]. Consequently, we observed an increase in the release of the apoptogenic component AIF, as well as the activation of executioner caspases. Our results demonstrate increased C-caspase 3 and C-PARP levels, further confirming the activation of caspase-dependent apoptosis. These pathways are frequently initiated by cellular stress or inflammation. In our study, the conditioned medium from HMC-3 microglia contained proinflammatory cytokines i.e., IL-6, IL-1β, and TNF-α and may also include other neurotoxic factors that cause neuronal injury. Consistent with our findings, Guadagno et al. found that microglia-derived TNF-α promotes neuronal death by upregulating the proapoptotic protein p53 upregulated modulator of apoptosis, which activates a Bax-dependent mitochondrial pathway [37].

Based on these findings, our in vivo results confirmed that mice exposed to poly (I:C) produce deleterious outcomes, especially in areas susceptible to inflammation. We examined working and spatial memory by evaluating spontaneous alternation using the T-maze test, which relies mainly on mice’s natural exploratory behavior in the absence of training, food, or water. Our results indicate a significant reduction in spontaneous alteration, suggesting working memory impairment. This impairment can be attributed to inflammatory response-led neurotoxicity, which interferes with hippocampal or prefrontal cortical signaling involved in spatial cognition and executive function [38]. Furthermore, the open field data showed a decreased exploratory behavior in poly (I:C)-treated mice, which is consistent with the study by Gibney et al. [39]. These results are consistent with anxiety-like behavior or a generalized sickness response, both of which are typical of the presence of systemic inflammation.

In contrast, there was no significant difference in rotarod and grip strength between the poly (I:C)-exposed mice and the control mice. This indicates that motor coordination and neuromuscular function remained intact after poly (I:C) exposure. These behavioral results are in line with the molecular analysis, where we observed an increase in gene expression of proinflammatory cytokines in the hippocampus but not in the cortex. In contrast, there is no evidence in the literature indicating that poly (I:C) specifically affects the hippocampus and not the cortex. Several studies have demonstrated that proinflammatory cytokines are more prominent in the hippocampus than in the cortex. For example, Porcher et al. observed age-related increases in cytokines, primarily in the hippocampus, compared to other brain regions, including the cortex [40]. Moreover, Shaker et al. found that the hippocampus is more susceptible to peripheral inflammatory stimuli [41].

Although the first study to examine the effect of poly (I:C) on human microglial cells and mice, the study has limitations that need to be addressed. First, in our in vivo model, we did not evaluate the long-term effects of poly (I:C) administration on neuroinflammation and cognitive outcomes. Additional studies are necessary to determine the sustained impact of poly (I:C) over time. Second, downstream signaling, such as oxidative stress, mitochondrial dysfunction, and microglial phagocytic behavior, was not examined. Third, the study was conducted only in male mice, which may restrict the generalizability of these findings. Addressing these limitations in future studies in addition to measuring the protein expression of key inflammatory mediators is recommended to fully understand the mechanisms by which poly (I:C) induces neuroinflammation, in a region-specific manner. Future studies should also validate the use of poly (I:C) as a reliable model for studying virus-induced neuroinflammation.

## Conclusion

**Fig. 7:**
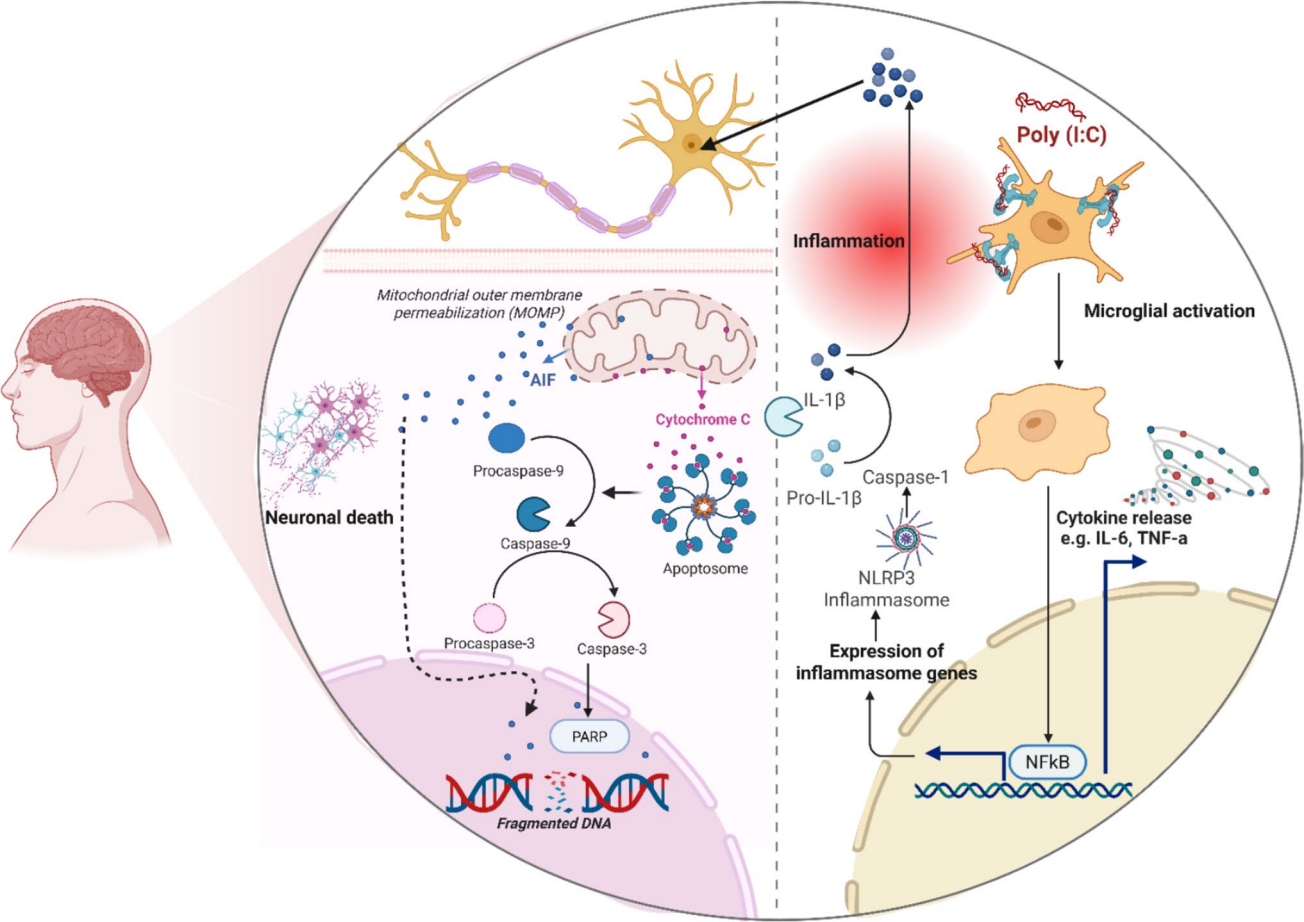
Poly (I:C) mechanism of microglia-activated neuronal apoptosis via NLRP3 inflammasome. Poly (I:C) binds to the TLR-3 on the microglia surface. This binding activates the intracellular NLRP3 inflammasome, which has a vital role in the cleavage and maturation of proinflammatory cytokines, specifically IL-1β and IL-18. These inflammatory cytokines act as signaling molecules, propagating the inflammatory response and directly impacting neighboring neurons. In neuronal cells, inflammatory signals trigger the intrinsic apoptotic pathway, which involves the sequential activation of executioner caspases (e.g., Cas-9 and Cas-3). The activation of these caspases leads to neuronal degeneration via apoptosis. Another key molecular mechanism of this apoptosis is the cleavage of PARP (poly-ADP ribose polymerase) into c-PARP. which is shown within the degenerating neurons as a definitive marker for apoptosis. Figure created using Biorender.com

The results of this study suggest that poly (I:C), a synthetic viral mimic, induces a robust inflammatory response in human microglial cells and mice. Poly (I:C) induces the release of increased proinflammatory cytokines and activates innate immune signaling pathways, such as NF-κB and NLRP3. These results highlight the reactivity of human microglia to viral-like stimuli and their crucial role in mediating neuroinflammation (Fig. 7). Furthermore, animals exposed to poly (I:C) developed region-specific neuroinflammatory responses, providing a valuable opportunity to understand the neuroinflammation associated with viral infections. Overall, this work established a crucial foundation for future studies aimed at elucidating the molecular mechanisms of neuroimmune activation and identifying potential therapeutic targets in virus-associated neurological diseases.

## Supplementary Information

Below is the link to the electronic supplementary material.

ESM 1 (DOCX 23.3 KB)

## Data Availability

Data will be made available on request.
