# Supplementary material for: Polyinosinic Polycytidylic Acid (poly I:C) Induces Neuronal Cell Death Through NF-κB-Mediated Inflammation in Human Microglia and Neuroinflammation-Induced Cognitive Impairment in Mice
Source: Mol Neurobiol. 2025 Nov 19;63(1):82. doi: 10.1007/s12035-025-05299-1 (PMC12627129; doi:10.1007/s12035-025-05299-1)
Supplement: Supplementary file 1 — (DOCX 23.3 KB) [file 12035_2025_5299_MOESM1_ESM.docx]

| **Table S1.Table of antibodies, vendor catalogue number, dilution factor by application, and lot number.** | | | |
| --- | --- | --- | --- |
| Antibody | Vendor/ Catalogue number | Host | Application/ Dilution |
| NLRP3 | CST/15101s | Rabbit | WB: 1:500 |
| IL-1β | CST/12242s | Mouse | WB: 1:500 |
| IL-6 | CST/12912s | Rabbit | WB: 1:500 |
| TNF-α | CST/8184s | Rabbit | WB: 1:500 |
| NF-κB | CST/8242s | Rabbit | WB: 1:1000  ICC: 0.7:1000 |
| Histone | CST/4499s | Rabbit | WB 1:1000 |
| Bad | CST/9239s | Rabbit | WB: 1:500 |
| Bax | CST/2772s | Rabbit | WB 1:500 |
| c-Caspase3 | CST/9664s | Rabbit | WB: 1:500 |
| Caspase-3 | CST/9662s | Rabboit | WB: 1:1000 |
| AIF | CST/5318s | Rabbit | WB: 1:500 |
| c-PARP | CST/5625s | Rabbit | WB: 1:500 |
| BCL2 | CST/3498s | Rabbit | WB: 1:1000 |
| Β-actin | Invitrogen/ MA1-140 | Mouse | WB: 1:1000 |
| Anti-mouse antibody | CST/7076s | Horse | WB: 1:1000 |
| Anti-rabbit antibody | CST/7074s | Goat | WB: 1:1000 |
| Alexa Fluor® | CST/4412 | Goat | ICC: 1:1000 |
